# Weighing the Risks: Small Bowel Obstruction Associated With Semaglutide Use in the Postoperative Abdomen

**DOI:** 10.7759/cureus.88589

**Published:** 2025-07-23

**Authors:** Natalie Nagib, Daniela Avila, Joanna Partyka, Armin Kamyab

**Affiliations:** 1 Medicine, Lake Erie College of Osteopathic Medicine, Bradenton, USA; 2 General Surgery, AdventHealth Sebring, Sebring, USA

**Keywords:** gastrointestinal motility, glp-1 receptor agonist, medical weight loss, postoperative adhesions, semaglutide, small bowel obstruction

## Abstract

Semaglutide, a glucagon-like peptide-1 (GLP-1) receptor agonist increasingly prescribed for weight loss, is generally well tolerated but may have underrecognized gastrointestinal complications. We present the case of a 59-year-old woman with an extensive abdominal surgical history who developed small bowel obstruction (SBO) shortly after semaglutide administration. Given semaglutide’s effects on gastric motility and its rising use, this case highlights the importance of caution when prescribing GLP-1 agonists to patients with a history of intra-abdominal surgeries and intra-abdominal adhesions.

## Introduction

Small bowel obstruction (SBO) can arise from a variety of mechanical or functional causes, each with important implications for clinical management and the potential need for timely surgical intervention [1]. Mechanical obstruction involves a physical blockage preventing the normal passage of intestinal contents and may be categorized by its anatomical origin: intraluminal (e.g., gallstone ileus), intramural (e.g., neoplastic lesions), or extrinsic (e.g., postoperative adhesions) [1]. In contrast, functional obstruction, also known as paralytic ileus, results from disrupted intestinal motility due to factors such as electrolyte imbalances or postoperative complications [1].

In developed countries, postoperative adhesions are the most common cause of mechanical SBO, contributing to approximately 74% of cases [1]. These fibrous bands often develop following abdominal or pelvic surgeries, such as appendectomies, colectomies, and gynecologic procedures, with adhesion formation reported in up to 97% of such operations [1].

Semaglutide is a glucagon-like peptide-1 (GLP-1) receptor agonist approved for glycemic control in type 2 diabetes mellitus and for reducing cardiovascular risk [2]. More recently, it has gained attention for its effectiveness in promoting weight loss in individuals with obesity [2]. Its therapeutic effects stem from GLP-1 receptor activation, which enhances insulin secretion, suppresses glucagon release, delays gastric emptying, and acts on the hypothalamus to reduce appetite and increase satiety [2].

While some studies have reported possible gastrointestinal side effects, GLP-1 receptor agonists are currently not strongly linked to an increased risk of SBO or ileus [3]. Among them, liraglutide has been associated with a slightly higher risk than semaglutide [3]. Although rarely acknowledged, case reports and emerging evidence suggest GLP-1 receptor agonists may contribute to bowel obstruction. In some regions, this potential risk remains absent from medication packaging and warning labels, leading to under-recognition in clinical practice [4].

## Case presentation

A 59-year-old female with a BMI of 40 presented with severe abdominal pain and bilious vomiting within hours of semaglutide administration. The patient was on semaglutide for a year but was unsure of the dosage as it was recently increased. She was unable to provide details regarding her regimen as she was receiving them in a non-clinical setting. The patient was not on any other medications affecting gastrointestinal motility. She reported approximately 15 episodes of vomiting within a few hours and presented directly to the emergency department due to the persistent vomiting. She endorsed nausea, bloating, and constipation. The patient denied prior SBO, including during her semaglutide regimen.

**Figure 1:**
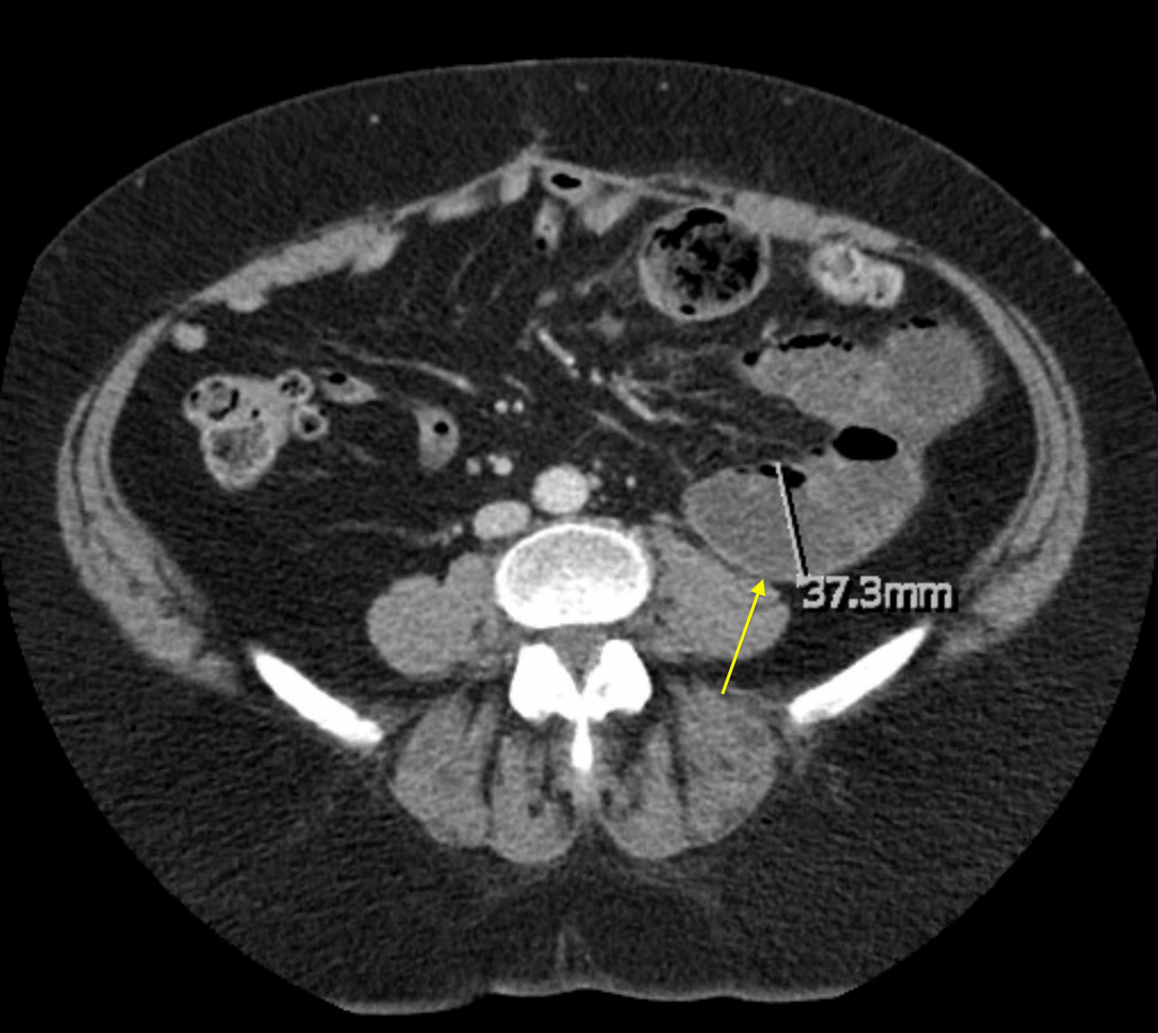
Axial CT scan shows prominent small bowel loops within the left upper and mid-abdomen measuring up to 3.7 cm (yellow arrow), consistent with SBO. CT: computed tomography; SBO: small bowel obstruction.

Her surgical history included colectomy for perforated diverticulitis, colostomy with reversal (12 years prior to SBO presentation), eight abdominal hernia repairs (seven years prior to SBO presentation), cholecystectomy, and appendectomy. Past medical history was otherwise unremarkable. She was afebrile and hemodynamically stable, with WBC of 13,000 cells/µL and lactic acid of 2.0 mmol/L. On abdominal physical exam, there was diffuse abdominal tenderness without guarding or rebound. No bruits were auscultated, and bowel sounds were high-pitched. A computed tomography (CT) scan revealed dilated small bowel loops up to 3.7 cm, suggestive of SBO (Figure 1).

**Figure 2:**
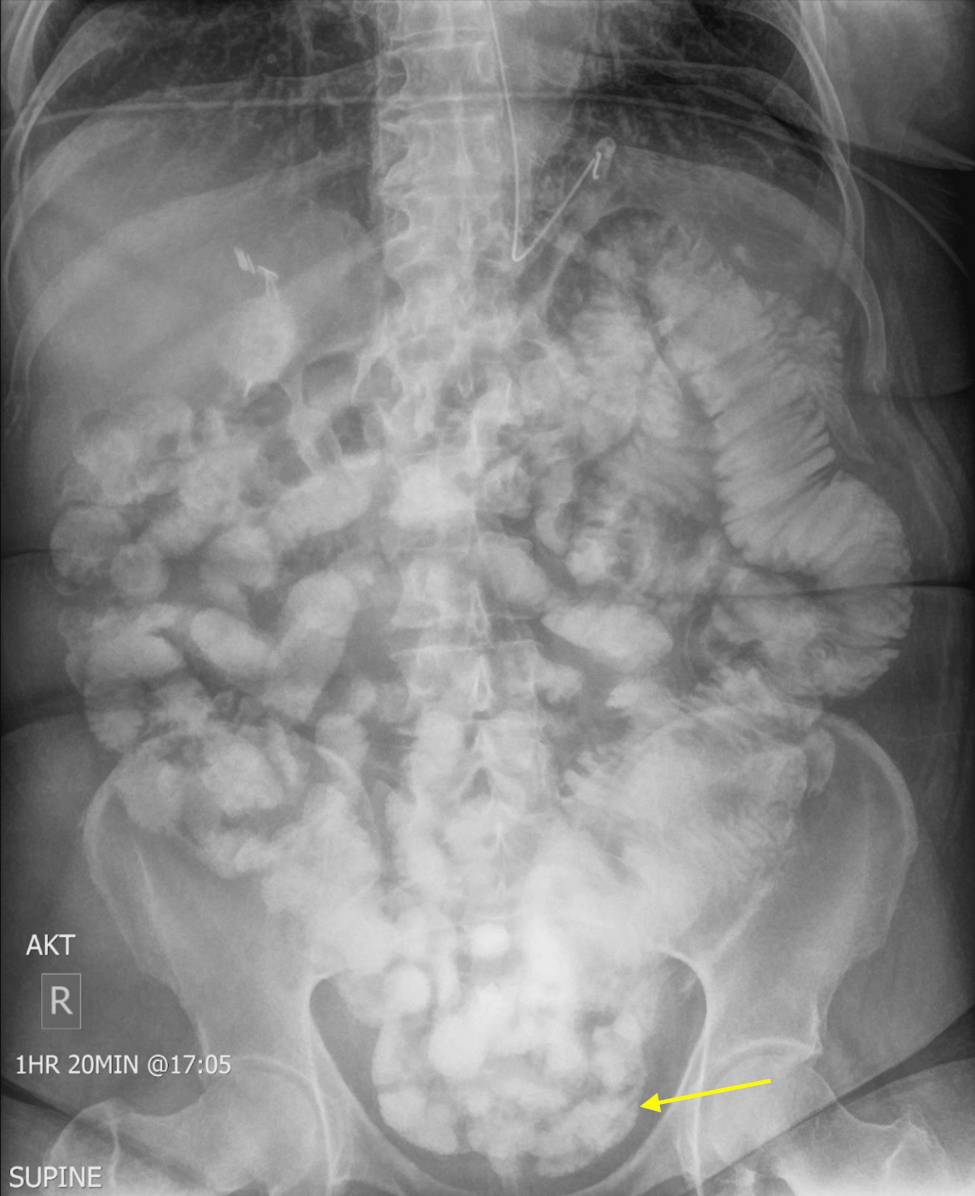
Small bowel series shows contrast passing to the colon (yellow arrow).

She was managed conservatively with bowel rest and nasogastric tube decompression. On hospital day 4, the patient reported complete resolution of abdominal pain, nausea, and vomiting. She was passing bowel movements and tolerating clear liquids. A small bowel follow-through with contrast showed passage of contrast into the colon after 90 minutes consistent with resolution of the SBO (Figure 2).

Her diet was advanced as tolerated, and by hospital day 5, she was discharged home. The patient was advised to follow-up outpatient with her primary care provider. The patient independently elected to discontinue semaglutide therapy following hospital discharge.

## Discussion

This case highlights the need for further investigation into whether GLP-1 receptor agonists, such as semaglutide, may contribute to gastrointestinal complications in patients with a history of abdominal surgeries. While a direct causal relationship cannot be established, this case raises awareness of a potential interaction warranting additional research and clinical vigilance. Postoperative adhesions are a well-established cause of mechanical SBO, particularly in patients who have undergone procedures such as colectomies and hernia repairs, such as in this patient [1]. The introduction of GLP-1 receptor agonists, such as semaglutide, may further complicate the clinical picture in this high-risk population.

Although the incidence of SBO associated with GLP-1 receptor agonists remains low, emerging case reports and basic science studies suggest a possible link [4]. These agents delay gastric emptying and modulate gastrointestinal motility, which may exacerbate pre-existing adhesive disease [2]. While semaglutide offers significant metabolic and cardiovascular benefits [2], this case seeks to raise awareness and caution of its use in patients with a known history of adhesions or previous abdominal surgery.

Given the widespread and increasing prescription of semaglutide for both diabetes and weight management, clinicians should maintain a high index of suspicion for gastrointestinal complications in patients presenting with abdominal pain, particularly those with a surgical history. Thorough history-taking, patient counseling, and close monitoring are essential. In some cases, alternative therapies with lower gastrointestinal risk profiles may be more appropriate. This case underscores the importance of informed decision-making and individualized risk assessment. Future research is needed to better characterize the potential relationship between GLP-1 receptor agonists and SBO, especially in patients with prior intra-abdominal surgery.

## Conclusions

In patients with prior abdominal surgeries, the risk of SBO due to postoperative adhesions is well known. This case raises questions of whether GLP-1 receptor agonists like semaglutide, which delay gastric emptying and alter gastrointestinal motility, may act as potential triggers for obstruction in such vulnerable individuals. Clinicians should thoroughly assess surgical history and counsel patients on this rare but significant potential complication before initiating semaglutide therapy. Individualized risk assessment is essential to ensure safe and effective use, especially as these agents become more widely prescribed.
